# Examining Integration of Comprehensive Substance Use Services in Certified Community Behavioral Health Clinics: A Cross-sectional Study

**DOI:** 10.1007/s10488-026-01515-9

**Published:** 2026-07-21

**Authors:** Yuanyuan Hu, Victoria Stanhope, Amanda I. Mauri, Houa Vang, Ran Hu

**Affiliations:** 1 School of Social Work, University of Minnesota, Minneapolis, USA; 2 Silver School of Social Work, New York University, New York, USA; 3 School of Public Health, University of Maryland, College Park, College Park, USA; 4 College of Social Work, The Ohio State University, Columbus, USA

**Keywords:** Certified community behavioral health clinics (CCBHC), Community mental health centers (CMHC), Substance use disorder (SUD) treatment

## Abstract

Despite policy efforts to expand integrated behavioral health care in the United States, substantial gaps remain in service delivery for people with co-occurring mental health (MH) and substance use disorders (SUD). Certified Community Behavioral Health Clinics (CCBHCs) are intended to close these gaps through enhanced financing and certification standards that mandate comprehensive, integrated MH–SUD services. Using the 2023 National Survey of Substance Use and Mental Health Services (N-SUMHSS) data for 1,480 facilities (198 CCBHCs, 448 community mental health centers [CMHCs], and 834 other outpatient MH facilities), this study compared service availability across three domains: engagement services, outpatient SUD treatment, and recovery support. Descriptive statistics characterized service provision, and multivariable logistic regressions estimated adjusted odds of service availability by facility type, controlling for ownership, government funding, and being a part of a multisite organization. Relative to other outpatient MH facilities, CCBHCs had significantly higher odds of providing engagement services, including interim services, outreach, and transportation, as well as outpatient SUD services, including outpatient detoxification and medication-assisted treatment. CCBHCs also exhibited greater availability of recovery-oriented support, including employment counseling, assistance obtaining social services, and housing supports. These findings provide some of the first national evidence that the CCBHC model is associated with substantially broader SUD-related service offerings than traditional CMHCs and other outpatient MH settings. By expanding both clinical SUD care and enabling services that reduce access barriers and address social determinants, CCBHCs appear well positioned to improve care continuity and recovery for individuals with co-occurring MH and SUD conditions.

## Introduction

People with co-occurring mental health and substance use disorders experience significantly higher morbidity and premature mortality, particularly among individuals with serious mental illness. Among individuals with schizophrenia, comorbid substance use disorders (SUDs) have been associated with approximately 50% to 100% higher odds of death (Lähteenvuo et al., 2021). In populations with opioid use disorder, co-occurring mental illness has been linked to higher mortality risk, increased emergency department visits and hospitalizations, and worse clinical course (Morin et al., 2020). However, the treatment gap for substance use disorder (SUD) service use is a pressing issue. The majority of individuals with SUD do not access treatment (Connery et al., 2020), with only 11.9% of individuals with past-year SUD reported receiving SUD treatment when pooling 2016–2019 data from the U.S. National Survey on Drug Use and Health (NSDUH) (Sahker et al., 2024). In 2023, approximately 48.5 million individuals over age 12 met diagnostic criteria for SUD, but only 14.6% received any specialist SUD treatment (Substance Abuse & Mental Health Services Administration [SAMHSA], 2024). The specialty treatment gap for SUD is even wider for low-income individuals, with 93% of adults with an income of less than 200% of the federal poverty level (FPL) not accessing treatment (Tomko et al., 2022). Among individuals with opioid use disorder (OUD), less than 30% accessed any form of OUD treatment, and around one-quarter received recommended medication-assisted treatment (Dowell et al., 2024; Saini et al., 2022). 41.2% of adults age 18 and older with co‑occurring mental health and substance use disorders, and 29.9% of adults with co‑occurring serious mental illness (SMI) and SUD received no treatment for either condition in the past year. On the service system level, key contributors to the treatment gap include limited access to SUD care, specialist programs, and clinicians' lack of skills and training (Connery et al., 2020).

One strategy demonstrated to decrease the treatment gap, called for by the Surgeon General, is to integrate comprehensive SUD services into mental health services (Office of the Surgeon General, 2016). Individuals who received mental health treatment in the past year were found to be more likely to receive SUD treatment (Sahker et al., 2024). Integrating SUD care into mental health settings contributed to higher detection, treatment uptake, and retention and improved patient outcomes, including increased treatment engagement, decreased substance use, and reduced psychiatric symptoms (Glover-Wright et al., 2023). Comprehensive SUD services that match individual clients' needs, including psychotherapy, medication-assisted treatment, recovery services, and physical care, can improve treatment access and engagement (Ramey et al., 2024). Such services were also associated with greater satisfaction with care; reduced relapse rates; and improved health, mental health, and social functioning (Cao et al., 2011; Hanson et al., 2019; Ramey et al., 2024).

Despite the benefits of integrating comprehensive SUD services into mental health settings, the likelihood of offering SUD care varies by the organizational characteristics of the mental health facility. Mental health organizations that were publicly owned, possessed state alcohol-and-drug licensure, operated dual-diagnosis programs, and received grant funding were more likely to deliver SUD services (Burns et al., 2023; Garrison et al., 2024). In states offering Medicaid waivers to increase co-occurring service provision, only private, for-profit facilities expanded co-occurring services, whereas public and nonprofit did not (Ge et al., 2024). Previous research has also found that outpatient behavioral health facilities that accept Medicaid, offer sliding-fee scales or other financial assistance, or operate within multisite systems were more likely to provide a broader range of SUD services (Edwards et al., 2014; Guerrero et al., 2014; Gutkind et al., 2025; Lindenfeld et al., 2024).

Certified Community Behavioral Health Clinics (CCBHCs) are federally supported behavioral health organizations that offer a comprehensive range of behavioral health services (National Council on Mental Health Wellbeing, 2024). Specifically, community mental health centers must fulfill SAMHSA criteria to receive the CCBHC designation that includes providing – directly or through other organizations – nine core services: outpatient mental-health and substance-use treatment, person- and family-centered treatment planning, community-based mental-health care for veterans, peer and family support with counselor services, targeted care management, outpatient primary-care screening and monitoring, psychiatric rehabilitation, and systematic screening, diagnosis, and risk assessment (SAMHSA, 2023). They must provide this care to anyone in need of services, regardless of their financial situation, residence, or age, and to people who are insured or uninsured, regardless of their ability to pay (Maeng et al., 2024). CCBHCs are also required to provide sliding scale discount schedules. The Section 223 Medicaid Demonstration, established under the Protecting Access to Medicare Act of 2014, created the framework for the initial CCBHC criteria and led to the designation of the 66 clinics across eight states in 2017–2018. Since then, the number of CCBHCs has expanded to over 500 across 46 states through expansions of the Section 223 Demonstration; other CMS approved, state Medicaid initiatives; and SAMHSA's Expansion Grant program (Substance Abuse and Mental Health Services Administration (SAMHSA), 2023).

Specific to SUD, CCBHCs must fulfill several requirements, including employing credentialed addiction specialists; providing medication used in the treatment of substance use; and offering comprehensive screenings, assessments, and timely interventions for SUDs (National Council for Behavioral Health, 2020). CCBHCs must maintain the capacity to deliver substance use disorder (SUD) treatment by employing licensed or certified addiction treatment staff and ensuring access to a medically trained behavioral health provider, either on staff or through a formal arrangement, who can prescribe and manage buprenorphine and other FDA‑approved medications for opioid, alcohol, and tobacco use disorders. Certification criteria further require the CCBHC to provide outpatient behavioral health services, including psychopharmacological treatment, either directly or through a Designated Collaborating Organization (DCO) agreement with another provider. Methadone treatment is excluded unless the CCBHC is also certified as an Opioid Treatment Program (OTP). Medications should specifically include buprenorphine and other medications used in treating opioid, alcohol, and tobacco use disorders (Centers for Medicare & Medicaid Services, 2024). CCBHCs also need to ensure access to comprehensive recovery-related services, either directly or through designated collaborating organizations (DCOs), such as psychiatric rehabilitation, peer support, counseling, family and caregiver support, and special recovery services tailored for veterans and active-duty military personnel (Centers for Medicare & Medicaid Services, 2024).

Because other community mental health centers are not required to fulfill these criteria, several studies have examined whether obtaining CCBHC status leads to improvements in SUD service offerings. A survey of CCBHCs found that 87% of CCBHCs provided at least one form of MOUD, higher than the national average of 64% among substance use treatment facilities (National Council for Mental Wellbeing, 2024). Additionally, other studies using data from comprehensive surveys of outpatient mental health treatment facilities found that CCBHCs were more likely to offer substance use disorder treatment services (Wishon & Brown, 2022), including medication for opioid use disorder (Cantor et al., 2024), than other organizations without a CCBHC designation. However, a 2022 secret shopper study found that, despite federal criteria requiring CCBHCs to provide MOUD on-site or through another organization, nearly one-third said they could neither offer nor refer patients for buprenorphine or methadone treatment (Presnall et al., 2022).

Shortcomings in existing CCBHC research weaken the strength and generalizability of these findings. Many studies employ descriptive analysis that do not adjust for potential confounders (Hu et al., 2021; Presnall et al., 2022; Wishon & Brown, 2022). Presnall and colleagues' secret shopper study (2022) faced several challenges, including that first-contact staff may not accurately represent available services and that data collection during the COVID-19 pandemic may have distorted service availability. Brown et al.'s (2023) analysis was limited to Medicaid beneficiaries already engaged in care before the demonstration and was based on a small number of states and programs. Similarly, Hu et al. (2024) focused narrowly on co-occurring disorder services rather than on the full continuum of substance use disorder care.

Our study addresses these gaps by assessing the extent to which CCBHCs integrate comprehensive substance use care compared to traditional community mental health centers (CMHCs) using nationwide data that allows for covariate adjustment. Comparing CCBHCs to CMHCs and other outpatient mental health clinics is essential because the CCBHC initiative was designed to transform the existing outpatient mental health safety-net sector through certification requirements and payment reform. The CCBHC criteria are designed to ensure that organizations that receive this designation offer comprehensive support to persons with a substance use disorder. It is important to empirically assess whether these requirements are associated with increased SUD service availability, such as medications for OUD and AUD and integrated care for co-occurring conditions, relative to CMHCs and other outpatient mental health clinics that are not required to meet CCBHC criteria. Doing so provides evidence on whether the CCBHC model translates regulatory requirements into meaningful real-world service expansion and improved availability of SUD service offerings (U.S. Government Accountability Office [GAO], 2021).

## Methods

### Data Sources

This study used data from the 2023 National Survey of Substance Use and Mental Health Services (N-SUMHSS) (SAMHSA, 2025) and conducted a cross-sectional, facility-level analysis. The N-SUMHSS is SAMHSA's annual census of U.S. behavioral-health treatment facilities, established in 2021 by merging the long-standing National Mental Health Services Survey (N-MHSS) and the National Survey of Substance Abuse Treatment Services (N-SSATS) instruments. All active providers in the Directories of Substance Use and Mental Health Treatment Facilities were invited to voluntarily respond to questions regarding facility characteristics, substance use and mental health treatment service offerings, special-population programs, and annual client counts (SAMHSA, 2025). The overall response rate among eligible facilities was 84.9% (SAMHSA, 2025). This study followed the Strengthening the Reporting of Observational Studies in Epidemiology (STROBE) reporting guidelines. Information bias may arise from self-reported service availability; N-SUMHSS uses standardized items administered uniformly across facility types to mitigate the bias.

### Primary Outcomes

We look at 12 SUD services that fall within three distinct domains that map onto the SUD treatment continuum: engagement services (e.g., outreach to persons in the community who may need treatment, interim services for clients when immediate admission is not possible, and transportation assistance to treatment), outpatient SUD treatment (e.g., intake and assessment, outpatient detoxification, medication-assisted treatment (MAT), intensive outpatient treatment, day treatment and standard outpatient treatment), and recovery and support services (e.g., employment counseling or training, assistance with obtaining social services, and assistance in locating housing). Each indicator was dichotomized: we coded each as 1 if a facility provided the service type and as 0 if the facility did not provide such a service.

### Treatment Facilities Covariates

We included types of outpatient mental health facilities as covariates, categorized into three groups: Certified Community Behavioral Health Clinics (CCBHC), Community Mental Health Centers (CMHC), and outpatient mental health facilities. We also adjusted for three additional facility characteristics: ownership (coded as private for-profit, 0; private nonprofit, 1; government-owned, 2); receipt of any direct government funding (coded as no, 0; yes, 1); and part of a multisite organization, operationalized as whether a facility was part of an organization with multiple substance use or mental health sites (coded as no, 0; yes, 1); whether the facility accepts Medicaid, and whether the facility accepts private insurance (coded as no, 0; yes, 1); whether the facility offer sliding fee scale (coded as no, 0; yes, 1).

### Data Analysis

We conducted descriptive statistics in STATA SE 19 and compared the proportions of service provision by the treatment facility covariates of facility type, ownership type, government funding, and part of a multisite organization using chi-square tests. For each of the 12 SUD-related service outcomes, multivariable logistic regression analyses were also conducted to estimate the odds ratio of service availability, also adjusting for the treatment facility covariates. Facilities with missing data on any analytic variable were excluded listwise.

## Results

Among the 1,480 community outpatient facilities that completed the substance-use services module, 198 (13.4%) were CCBHCs, 448 (30.3%) were Community Mental Health Centers (CMHCs), and 834 (56.4%) were other outpatient mental health facilities without either designation. In terms of ownership, 374 facilities (25.3%) were private, nonprofit; 899 (60.7%) were private, for-profit; and 207 (14.0%) were publicly operated. The majority of facilities reported receiving direct government funding (1,008, 68.1%) and belonged to multisite systems (1,234, 83.4%). 1387 (93.72%) facilities accepted Medicaid, and 1339 (90.47%) accepted private insurance. 1216 (82.16%) facilities used sliding scale fee (See Table 1).

**Table 1:** Descriptives of SUD service provision

Outcome	Intake assessment	Day treatment	Detox services	MAT services	Intensive treatment	Standard treatment	Transportation services	Employment services	Outreach services	Interim services	Social services	Finding housing
	N (%)	N (%)	N (%)	N (%)	N (%)	N (%)	N (%)	N (%)	N (%)	N (%)	N (%)	N (%)
All (N = 1480)	1470 (99.32)	126 (8.51)	120 (8.11)	806 (54.46)	693 (46.82)	1,455 (98.31)	754 (50.95)	723 (48.85)	1,141 (77.09)	978 (66.08)	1,171 (79.12)	1055(71.28)
Outpatient MH (N = 834)	827 (99.2)	86 (10.3)	66 (7.9)	425 (51)	407 (48.8)	812 (97.4)	345 (41.4)	330 (39.6)	589 (70.6)	485 (58.2)	573 (68.7)	505 (47.87)
CCBHC (N = 198)	196 (99)	8 (4)	24 (12.1)	134 (67.7)	93 (47)	198 (100)	142 (71.7)	146 (73.7)	178 (89.9)	160 (80.8)	183 (92.4)	174 (16.49)
CMHC (N = 448)	447 (99.8)	32 (7.1)	30 (6.7)	247 (55.1)	193 (43.1)	445 (99.3)	267 (59.6)	247 (55.1)	374 (83.5)	333 (74.3)	415 (92.6)	376 (35.64)
Private nonprofit (N = 374)	371 (99.2)	70 (18.7)	48 (12.8)	191 (51.1)	199 (53.2)	358 (95.7)	119 (31.8)	140 (37.4)	258 (69)	205 (54.8)	218 (58.3)	680 (64.45)
Private for profit (N = 899)	893 (99.3)	46 (5.1)	52 (5.8)	512 (57)	392 (43.6)	892 (99.2)	490 (54.5)	471 (52.4)	697 (77.5)	607 (67.5)	769 (85.5)	200 (18.96)
Government ownership (N = 207)	206 (99.5)	10 (4.8)	20 (9.7)	103 (49.8)	102 (49.3)	205 (99)	145 (70)	112 (54.1)	186 (89.9)	166 (80.2)	184 (88.9)	175 (16.59)
Gov Funding (N = 1008)	1,002 (99.4)	57 (5.7)	63 (6.2)	581 (57.6)	488 (48.4)	996 (98.8)	593 (58.8)	530 (52.6)	837 (83)	735 (72.9)	892 (88.5)	807 (76.49)
Multiple facilities (N = 1245)	1,236 (99.3)	92 (7.4)	95 (7.6)	706 (56.7)	570 (45.8)	1,223 (98.2)	657 (52.8)	632 (50.8)	975 (78.3)	832 (66.8)	1,004 (80.6)	918 (87.01)
Accepting medicaid	1,379 (93.81%)	101 (80.16%)	101(84.17)	765 (94.91%)	651 (93.94%)	1,370 (94.16%)	740 (98.14%)	700 (96.82)	1,077 (94.39)	932 (95.30)	1,147 (97.95)	1,034 (98.01)
Accepting private insurance	1,332 (90.61%)	117 (92.86%)	113 (94.17)	752 (93.30%)	613 (88.46%)	1,316 (90.45%)	661 (87.67%)	633 (87.55%)	1,034 (90.62)	884 (90.39)	1,053 (89.92)	947 (89.76)
Sliding scale Fee	1,209 (82.24%)	99 (78.57%)	90(75)	667 (82.75%)	564 (81.39%)	1,202 (82.61%)	660 (87.53%)	619 (85.62%)	976 (85.54)	834 (85.28)	1,000 (85.40)	907 (85.97)

### Delivery of SUD Services

There were differences in the availability of outpatient SUD treatment across the 1,480 community mental health programs. Specifically, many facilities conducted standardized substance use intake assessments (1,470, 99.32%) and delivered standard outpatient substance use treatment (1,455, 98.31%). However, more specialized outpatient substance use services were less common, with MAT available in 806 (54.46%) sites, intensive outpatient services in 693 (47%), and day treatment services available in only 126 (8.51%) sites.

A large majority of programs provided engagement services: 1,141 programs (77.1%) provided outreach to individuals in the community who may need treatment, and 978 facilities (66.1%) offered interim or "bridge" services, including contact and counseling, for clients when immediate admission is not possible. Approximately half of the facilities (51.0%, n = 754) provided transportation services for clients.

In terms of recovery services, 723 (48.9%) facilities offer employment counseling or training, 1,171 (79.1%) facilities assisted clients in obtaining social service benefits, and 1,055 facilities (71.3%) helped clients locate stable housing.

### Facility Type Comparisons

After adjustment for ownership, government funding, and part of a multisite organization, logistic regression results demonstrated that compared to the reference group of other outpatient mental health facilities, CCBHCs have a higher adjusted odds ratio of delivering key engagement services, including interim services (aOR = 2.46, p < 0.01), outreach (aOR = 2.89, p < 0.01), and transportation services (aOR = 2.43, p < 0.01) (See Table 2). CCBHCs were also significantly more likely to offer outpatient SUD treatment services, including outpatient detoxification (aOR = 3.55, p < 0.01) and MAT (aOR = 1.87, p < 0.01) and all kinds of recovery support services, including employment, counseling or training (aOR = 3.73, p < 0.01), assistance with obtaining social services (aOR = 3.00, p < 0.01), and housing support (aOR = 3.08, p < 0.01). Compared to other outpatient mental health facilities, CMHCs had higher odds of offering outpatient detoxification (aOR = 1.74, p = 0.047), while differences for other outpatient SUD treatment modalities were not statistically significant. they had higher odds of delivering engagement services (e.g., interim services (aOR = 1.66, p < 0.01), outreach (aOR = 1.60, p < 0.01), transportation (aOR = 1.51,p < 0.01), and recovery supports (e.g., employment counseling (aOR = 1.70, p < 0.01), social services (aOR = 3.10, p < 0.01), housing (aOR = 2.27, p < 0.01)).

**Table 2:** Logistic regression results

Outcome	Intake assessment	Day treatment	Detox services	MAT services	Intensive treatment	Standard treatment	Transportation services	Employment services	Outreach services	Interim services	Social services	Finding housing
	aOR (95%CI)	aOR (95%CI)	aOR (95%CI)	aOR (95%CI)	aOR (95%CI)	aOR (95%CI)	aOR (95%CI)	aOR (95%CI)	aOR (95%CI)	aOR (95%CI)	aOR (95%CI)	aOR (95%CI)
CCBHC**	0.77 (0.14, 4.32)	0.92 (0.41, 2.09)	**3.55 (1.94, 6.49)**	1.87 (1.31, 2.66)	0.99 (0.71, 1.39)	NA*	**2.43 (1.69, 3.50)**	**3.73 (2.58, 5.40)**	**2.89 (1.73, 4.82)**	**2.46 (1.64, 3.69)**	**3.00 (1.69, 5.35)**	**3.08 (1.92, 4.96)**
	0.769	0.850	** < 0.001**	**0.001**	0.959	NA*	** < 0.001**	** < 0.001**	** < 0.001**	** < 0.001**	** < 0.001**	** < 0.001**
CMHC**	3.22 (0.35, 30.02)	1.52 (0.90, 2.57)	**1.74 (1.01, 3.02)**	1.06 (0.81, 1.37)	0.82 (0.63, 1.07)	1.96 (0.50, 7.76)	**1.46 (1.12, 1.91)**	**1.70 (1.31, 2.21)**	**1.60 (1.15, 2.23)**	**1.66 (1.24, 2.20)**	**3.10 (2.04, 4.71)**	**2.27 (1.65, 3.13)**
	0.304	0.119	**0.047**	0.680	0.137	0.335	**0.005**	** < 0.001**	**0.005**	**0.001**	** < 0.001**	** < 0.001**
Private nonprofit	0.79 (0.13, 4.99)	**0.26 (0.15, 0.46)**	**0.49 (0.27, 0.89)**	0.80 (0.58, 1.10)	**0.47 (0.34, 0.66)**	**4.11 (1.26, 13.39)**	1.22 (0.87, 1.70)	1.08 (0.78, 1.50)	**0.60 (0.42, 0.86)**	0.77 (0.55, 1.07)	1.23 (0.85, 1.78)	0.86 (0.61, 1.21)
	0.807	** < 0.001**	**0.020**	0.171	** < 0.001**	**0.019**	0.248	0.640	**0.005**	0.121	0.279	0.384
Government ownership	1.22 (0.08, 17.58)	**0.25 (0.11, 0.56)**	0.97 (0.47, 2.02)	**0.60 (0.40, 0.91)**	**0.52 (0.34, 0.80)**	3.46 (0.63, 18.83)	**2.41 (1.56, 3.74)**	1.23 (0.81, 1.88)	1.50 (0.85, 2.66)	1.43 (0.90, 2.29)	1.46 (0.83, 2.58)	1.53 (0.92, 2.54)
	0.884	**0.001**	0.933	**0.017**	**0.003**	0.151	** < 0.001**	0.337	0.165	0.130	0.191	0.100
Gov funding	0.94 (0.17, 5.23)	0.84 (0.49, 1.43)	0.58 (0.34, 1.00)	**1.58 (1.18, 2.13)**	**2.02 (1.48, 2.74)**	0.38 (0.12, 1.18)	**1.46 (1.08, 1.97)**	0.94 (0.69, 1.26)	**2.20 (1.58, 3.08)**	**1.98 (1.46, 2.68)**	**2.38 (1.67, 3.40)**	**1.92 (1.39, 2.65)**
	0.941	0.513	0.052	**0.002**	** < 0.001**	0.093	**0.014**	0.667	** < 0.001**	** < 0.001**	** < 0.001**	** < 0.001**
Multiple facilities	0.42 (0.05, 3.66)	0.69 (0.44, 1.08)	0.83 (0.51, 1.36)	**1.55 (1.15, 2.08)**	0.80 (0.60, 1.07)	0.43 (0.12, 1.51)	1.26 (0.92, 1.72)	1.34 (0.99, 1.81)	1.31 (0.94, 1.84)	1.01 (0.74, 1.38)	1.05 (0.72, 1.51)	**1.52 (1.10, 2.10)**
	0.434	0.106	0.470	**0.003**	0.131	0.188	0.145	0.060	0.109	0.930	0.804	**0.011**
Accepting medicaid	3.38 (0.60, 19.14)	**0.37 (0.21, 0.66)**	**0.41 (0.22, 0.75)**	1.17 (0.74, 1.85)	1.21 (0.77, 1.91)	4.93 (1.74, 13.94)	**4.02 (2.13, 7.56)**	**2.34 (1.39, 3.94)**	0.80 (0.49, 1.32)	1.17 (0.74, 1.85)	**6.57 (3.83, 11.27)**	**5.45 (3.16, 9.40)**
	0.168	**0.001**	**0.004**	0.501	0.414	0.003	** < 0.001**	**0.001**	0.384	0.495	** < 0.001**	** < 0.001**
Accepting private insurance	3.65 (0.89, 14.96)	1.58 (0.76, 3.30)	2.17 (0.97, 4.84)	**2.07 (1.44, 2.99)**	**0.64 (0.45, 0.92)**	0.81 (0.18, 3.59)	0.95 (0.62, 1.46)	0.89 (0.61, 1.31)	**0.50 (0.29, 0.84)**	**0.53 (0.34, 0.84)**	0.95 (0.62, 1.46)	0.89 (0.61, 1.31)
	0.072	0.223	0.059	** < 0.001**	**0.015**	0.784	0.808	0.565	**0.010**	**0.007**	0.808	0.565
Sliding scale Fee	1.36 (0.30, 6.10)	1.53 (0.92, 2.54)	0.76 (0.46, 1.26)	0.83 (0.62, 1.12)	0.98 (0.73, 1.32)	1.86 (0.76, 4.56)	1.34 (0.97, 1.84)	1.15 (0.84, 1.56)	**1.68 (1.22, 2.31)**	1.16 (0.86, 1.57)	1.04 (0.74, 1.48)	1.19 (0.86, 1.64)
	0.690	0.099	0.289	0.233	0.887	0.173	0.074	0.386	**0.002**	0.335	0.807	0.299

Bolded values indicate statistically significant results. Statistical significance was defined as p < .05

*All surveyed CCBHCs provided standardtreatment and did not have variation

**Reference group is other outpatient mental health facilities

Other facility-level characteristics, including ownership type, funding sources, and facility size, were also found to be significantly associated with the provision of key engagement, outpatient treatment, and recovery support. Compared with private-for-profit facilities, private not-for-profit facilities were more likely to provide standard treatment (aOR = 4.11, p < 0.05) and outreach services (aOR = 0.60, p < 0.05) but less likely to offer day treatment (aOR = 0.26, p < 0.01), detoxification services (aOR = 0.49, p < 0.05), and intensive outpatient services (aOR = 0.47, p < 0.01). On the other hand, government-owned facilities, compared with private-for-profit facilities, had higher odds of offering transportation services (aOR = 2.71, p < 0.01), and assistance with finding housing (aOR = 1.81, p < 0.05). Such facilities had lower odds of day treatment (aOR = 0.25, p < 0.01), MAT services (aOR = 0.59, p < 0.01), and intensive treatment (aOR = 0.35, p < 0.01).

Receipt of direct government funding was associated with higher odds of providing MAT services (aOR = 1.58, p < 0.01), intensive treatment (aOR = 2.02, p < 0.01), all engagement services (transportation services (aOR = 1.46, p < 0.01), outreach services (aOR = 2.20, p < 0.01), interim services (aOR = 1.98, p < 0.01)), and two types of recovery services, including social services (aOR = 2.38, p < 0.01) and assistance with finding housing (aOR = 1.92, p < 0.01). Facilities that were part of a multiple-site facility had higher odds of providing medication-assisted treatment (MAT) services (aOR = 1.55, p < 0.01) and assistance with finding housing (aOR = 1.52, p < 0.05). Facilities accepting Medicaid had higher odds of providing transportation services (aOR = 4.02, p < 0.01), employment services (aOR = 2.34, p < 0.01), social services assistance (aOR = 6.57, p < 0.01), and housing support (aOR = 5.45, p < 0.01), and were also more likely to offer standard treatment (aOR = 4.93, p < 0.01). In contrast, Medicaid acceptance was associated with lower odds of offering day treatment (aOR = 0.37, p < 0.01) and detox services (aOR = 0.41, p < 0.01). Accepting private insurance was associated with higher odds of offering MAT services (aOR = 2.07, p < 0.001), but lower odds of providing intensive treatment (aOR = 0.64, p < 0.05), transportation services (aOR = 0.38, p < 0.01), employment services (aOR = 0.43, p < 0.01), social services assistance (aOR = 0.50, p < 0.05), and housing support (aOR = 0.53, p < 0.01). Offering a sliding-scale fee was also associated with higher odds of providing outreach services (aOR = 1.68, p = 0.002).

## Discussion

These findings are among the first to use national data to directly compare CCBHCs, CMHCs, and other outpatient MH facilities in their provision of SUD treatment. Previous research has focused on descriptive analysis and did not provide details on SUD services (Wishon & Brown, 2022), regional systems (Brown et al., 2023), or had a restricted sample (Hu et al., 2021), which limited the ability to compare CCBHCs, CMHCs, and other outpatient mental health facilities. By utilizing a national dataset, N-SUMHSS, this study found that nationally, CCBHCs were more likely to provide a wide range of engagement services, outpatient SUD treatment, and recovery services, indicating that CCBHCs are more likely to offer a continuum of SUD services compared to other outpatient community mental health providers.

The increased service availability in CCBHCs suggests that this model may meet one of its primary goals of expanding outpatient SUD care. The CCBHC model is designed to transform outpatient mental health clinics, including CMHCs, into integrated, comprehensive behavioral health providers through standardized certification requirements and enhanced financing. Comparisons with CMHCs and other outpatient mental health facilities are of policy significance to evaluate whether the model is achieving its intended service integration goals (Breslau et al., 2016; Wishon & Brown, 2023). Notably, CCBHCs were almost twice as likely to provide medication used in the treatment of substance use disorders as other outpatient mental health organizations. The potential of CCBHCs to address the opioid epidemic garnered much political support during initial legislative debates about creating the model, and our findings indicate that CCBHCs may be fulfilling this potential (National Council for Mental Wellbeing, 2022). These findings provide empirical support that the combination of CCBHC certification requirements for minimum services and financial enhancement to supply the resources to staff, operationalize, and sustain SUD workflows and pharmacotherapy capacity is associated with more comprehensive SUD service capacity in outpatient behavioral health settings (Breslau et al., 2016; Brown et al., 2023; Hu et al., 2024). However, the fact that a little more than half of outpatient mental health facilities delivered MOUD shows that much more needs to be done to meet the needs of people with OUD.

Key to the findings was not that CCBHC just expanded access to SUD but that they provide comprehensive care. Previous studies identified that barriers to SUD treatment are multidimensional and that fragmented care systems lead to significant treatment gaps (Farhoudian et al., 2022; Tomko et al., 2022). To address, this issue, it is essential to establish a continuum of care that encompasses initial engagement, treatment, and recovery services (McCance-Katz, 2025; Ussery et al., 2025). Research shows that improving engagement services, including outreach, contact while on the waitlist, and transportation assistance, reduces barriers to care (Ellerbe et al., 2017) and improves treatment initiation (Scott et al., 2018; Stanojlović & Davidson, 2021). Recovery services, including employment counseling or training, assistance with obtaining social services, and assistance in locating housing, were found effective in reducing relapse rates, improving treatment retention, and enhancing social connections and social functioning (Reif et al., 2014; Sahin & Elboga, 2019; Vanderplasschen et al., 2019).

A clearly defined care continuum, supported by integrated care models such as Certified Community Behavioral Health Clinics (CCBHCs), promotes consistent support across critical stages of SUD services, potentially facilitating long-term recovery and improving overall health outcomes (McCance-Katz, 2025; Ussery et al., 2025). As previous research has underscored, insurance expansion alone is insufficient to close the SUD treatment gap (Tomko et al., 2022). Our findings reinforce the recommendation to expand CCBHCs as a model that proactively addresses multidimensional barriers to recovery and establishes a continuum of care.

Our study results also discovered relationships between organizational characteristics and service provision. In line with prior work (Burns et al., 2023; Garrison et al., 2024), we found that facilities were more likely to provide comprehensive SUD services when they received direct government funding, were publicly or nonprofit owned, and operated as part of a multisite system. Facilities accepting Medicaid were more likely to offer a more comprehensive array of supportive and recovery services. A higher proportion of CCBHCs reported these characteristics relative to non-CCBHC providers, underscoring that organizational features, including receipt of direct government funding, public or nonprofit ownership, and being part of multisite systems, are tied to a greater likelihood of being designated as a CCBHC. These patterns also suggest that public-supported models, through mechanisms such as governmental funding, rather than profit-oriented models, are essential for integrating comprehensive SUD services into outpatient mental health care.

### Limitations

This study has several limitations. First, a key limitation is the inability to determine whether CCBHCs were providing more comprehensive SUD services prior to certification, or whether such services were newly introduced in response to CCBHC requirements. The findings should be interpreted as CCBHC status may lead to expanded services, but further studies are needed to identify any causal effect that CCBHC status led to an increase in SUD offerings. Second, indicators for service availability rely on self-reported data without external validation, which may pose risks of inaccuracies, especially for newly implemented SUD services, such as medication-assisted treatment. Third, the cross-sectional design of this study limits the capacity to establish causal inferences between facility type and service availability. Future studies may compile multiple years of data and employ trend analysis methods to evaluate changes in SUD service availability over the years. Finally, this study focuses solely on service availability due to the lack of client-level health outcomes. Because N‑SUMHSS is a facility survey focused on self-reported, on-site service availability, our measures primarily capture the comprehensiveness of SUD-related services, and we operationalize service integration based on the reported availability of SUD services rather than patient-level service receipt. Further research is needed to investigate whether CCBHCs can more effectively treat individuals with co-occurring conditions by improving access to services. Additional analyses should leverage EHR or claim data, linked with geospatial information, to evaluate integrated service receipt and access across clinic types.

## Conclusion

This study highlights the critical role that Certified Community Behavioral Health Clinics (CCBHCs) play in providing comprehensive SUD services. CCBHCs offer more comprehensive SUD services, covering engagement, treatment, and recovery domains, than other community mental health providers. These findings suggest that public investment in the CCBHCs initiative represents a potential strategy for addressing persistent gaps in building a continuum of SUD care. Future policy efforts should continue to expand the CCBHC model as a national effort to deliver equitable, accessible, and effective substance use treatment.

## Data Availability

This study uses secondary data from the 2023 National Survey of Substance Use and Mental Health Services (N-SUMHSS), collected by the Substance Abuse and Mental Health Services Administration (SAMHSA). The dataset is publicly available and can be accessed through the SAMHDA (Substance Abuse and Mental Health Data Archive) at: https://www.datafiles.samhsa.gov.
